# Book Reviews

**Published:** 1874-04-01

**Authors:** 


### Book Reviews.

A CLINICAL History of the Medical and Surgical Diseases of Women. By Robert Barnes, M.D., London, Fellow and Lumleian Lecturer (1873) Royal College of Physicians; Examiner in Obstetrics and the Diseases of Women, at the University of London and the Royal College of Surgeons; Obstetric Physician, and Lecturer on Obstetrics and Diseases of Women, to St. Thomas' Hospital. Philadelphia: Henry C. Lea, 1874.

We gladly welcome this new work on Diseases of Women, by Dr. Barnes, who has long been considered high authority in this branch of medicine. The author states that the design of the work is not so much for the specialist, as "to give such a description of the medical and surgical diseases of women as will assist the medical practitioner in their diagnosis and treatment." Each subject, however, seems to be carefully and adequately discussed; and the introductory chapters, on the minute anatomy as applied to the sexual system, are exceedingly full and valuable. One chapter contains a description of different gynæcologocal instruments, in which we notice nothing new; and the remainder of the book is devoted to the diagnosis, pathology, and treatment of female diseases proper. The text is handsomely illustrated, and the engravings are nearly all new, not copied from other works which have long been familiar to us, but taken from actual pathological specimens, preserved in the museums of the London hospitals.

Among other new things, the author has introduced many new words of Greek origin, which he proposes as new names for some diseases, because, as he believes, they express more accurately their description, or pathology. These new names may be more scientific, or more classical, yet we are inclined to be partial to the old ones.

As regards treatment, it is noticeable how frequently Dr. Barnes recommends the use of the pessary as a means of bringing about a cure. He says: "If pessaries are found useful, it matters little whether they satisfy the conditions of science. That thousands of women find comfort and benefit from their use, is a fact too notorious to be disputed. Still, it is asserted that their usefulness, being only palliative, and temporary, and science supplying modes of treatment which are curative, pessaries should be discarded. If the premises are true, we could not reject the conclusion. But they are not true; and a wide field is still left for study, and the application of various modes of treatment, according to the various forms of the malady." Undoubtedly, many women have found comfort and benefit from their use, yet it is the experience of many gynæcologists that as many women, if not more, have received decided harm and injury from them. How often does it happen that a physician introduces a pessary, perhaps intending it only as a palliative, and temporary treatment, and the patient goes away half comprehending what is intended, and does not return till the instrument has produced a state of things worse than the original disease? Too many worthless, if not injurious, pessaries, are being offered to the profession; and the least objectionable ones, in our opinion, should only be used as a last resort, when all curative measures have failed to meet the circumstances of the case.

Tumors of the uterus are treated at length, including the latest investigations, and many practical points are suggested. Cancer of the uterus has received the attention which the importance of the subject demands. In a word, the treatise is thoroughly practical, drawn from a rich personal experience, and is entitled to a place among the leading works on diseases of women. W. H. W.

---

The Chicago Journal of Nervous and Mental Disease. Edited by J. S. Jewell, M.D., and H. M. Bannister, M.D., January, 1874.

The first number of this new periodical is before us, containing 114 neatly printed pages; and is filled with a good variety of most interesting articles. The first of these is a lecture on "The Pathology of the Vaso-Motor Nervous System," occupying seventeen pages, by the senior editor. Another article, occupying fourteen pages, is an abstract of a lecture at St. Petersburgh, by E. Cyon, "On the Relations of the Heart's Action to the Nervous System, and to Mental and Emotional States." These two leading articles are worth more than the price of the *Journal* for one year. But the editors, by translations, abstracts, and selections, have filled every page of this number with matter of interest and value. From what we know of the ability and industry of the editors and publishers, we assure our readers that they will find it all that it claims to be. It will be published quarterly, at $4.00 per annum; single copies, $1.00. Address, the editors, 57 Washington street, Chicago. N. S. D.

Annual Report of the Supervising Surgeon of the Marine Hospital Service of the United States, for the Fiscal Year 1873 (July 1, 1872 to July 30, 1873). By John M. Woodworth, M.D. Washington: Government Printing Office, 1873. pp. 154.

Dr. Woodworth has certainly accomplished much in the way of reform in the Marine Hospital Service, by his energy and untiring perseverance; and is fairly entitled to the congratulations of the profession. The present is the second of his official reports which have been printed for distribution, and it bears internal evidence of improvement in the general management and construction of hospitals, and their recent direction to scientific methods of examination, treatment, and record. Appendix "B," of this report, "On the Natural History of Yellow Fever in the United States," is from the pen of Dr. J. M. Toner, of Washington, so favorably known in connection with the foundation of the Toner Lectures. It is illustrated by a chart of elevations above the sea-level, of localities where the disease has prevailed. This is exceedingly valuable as an aid to the study of the ravages of yellow fever upon this continent.

The Supervising Surgeon has a grand field before him; but he has, also, a duty to perform, which, if it be not well discharged, will almost neutralize his other achievements—it is the complete separation of the Marine Hospital Service of the United States from all political control. Science, when shadowed by the upas-tree of politics, is a dwarfed and defenceless object of pity. Dr. Woodworth understands this; and we unite with all who wish him success in his efforts to uproot the gigantic evil. "*Macte virtute esto.*" J. N. H.

Lectures on the Clinical Uses of Electricity, Delivered in the University College Hospital. By J. Russell Reynolds, M.D., F. R.S., Fellow of the Royal College of Physicians, etc. Second Edition. Philadelphia: Lindsay & Blakiston, 1874. pp. 118.

Galvano-Therapeutics; a Revised Reprint of a Report made to the Illinois State Medical Society, 1873. Philadelphia: Lindsay & Blakiston, 1873. pp. 64.

The therapeutic uses of electricity and galvanism are little understood by general practitioners of medicine; and those who have specially investigated the subject are far from being in accord as to the methods of treatment, and the diseases in which such treatment is indicated. That volume has yet to be written which will, on the one hand, satisfy the actual needs of the profession, and, on the other, be fully received as an authority by those who are in the advance of electro-galvanic science. As an *avant-courier* of this book of the future, each of the volumes before us deserves the attention of those who desire to be familiar with the literature of the subject.

As regards the subject of electricity and galvanism in cutaneous disorders—a field recently developed by the researches of Beard, of New York, and which, judging from his published papers, promises abundant harvests for its explorer—we note that the English author is entirely silent, and the American has but little to offer. This fact is an index of the relative practical value of the two volumes.

---

The Nature of Gunshot Wounds of the Abdomen, and their Treatment; based on a Review of the Case of the Late James Fisk, Jr., in its Medico-Legal Aspects. By Eugene Peugnet, M.D., Surgeon to the Northwestern Dispensary, &c. New York: Wm. Wood & Co., 1874. pp. 96.

A scholarly and well-written monograph, fortified at every point by authoritative citations, and lucid in every detail, is rather a matter of surprise in these days of loose authorship. Dr. Willard Parker may well feel complimented by the dedication to him of this labor of his former pupil.

One cannot avoid concurring with the author, after a perusal of his premisses, in the following conclusions, deduced from the medical jurisprudence of this *cause celebre:*

1. The shooting of Fisk was not done in self-defence; but with premeditation.

2. The abdominal wound was not necessarily fatal; and the morphia administered was the immediate cause of death.

"He who profits by his blunders is least likely to commit them."

---

A Handbook of Medical and Surgical Reference. By John A. Wyeth, M.D., Member of the New York Pathological Society, &c. New York: Wm. Wood & Co. 1873. pp. 279.

This little volume is intended, mainly, as the author declares in its introduction, "to assist the young practitioner in his labors." It contains information which should properly be stored, in large part, in the minds of old and young practitioners alike. But if this were the case, the usefulness of the little manual might be impaired, and so we may be sure, in either case, the information is available. The book is bound with a fold for convenience of the pocket.

